# Stability of financial development: Problems of measurement, assessment and regulation

**DOI:** 10.1371/journal.pone.0277610

**Published:** 2022-11-21

**Authors:** Galina G. Gospodarchuk, Elena S. Zeleneva

**Affiliations:** 1 Department of Finance and Credit, National Research Lobachevsky State University of Nizhny Novgorod, Nizhny Novgorod, Russia; 2 Department of Banking and Monetary Regulation, Financial University under the Government of the Russian Federation, Moscow, Russia; Szechenyi Istvan University: Szechenyi Istvan Egyetem, HUNGARY

## Abstract

*Adequate measurement*, *assessment*, *and regulation of financial development stability are key components*, *the formation of effective macroprudential policies*, *and the coordination of these policies among countries*. *However*, *an analysis materials on the subject shows that work in this area is fragmented*,. *In this regard*, *the purpose of this study is to develop a* methodology for diagnosing and regulating the stability of financial development based on a systematic approach. The study used systematic, comparative, and GEO analysis, econometric methods. As a result, new indicators have been developed to diagnose the financial development of countries. The authors also propose criteria, which could be used to signal the need for countercyclical regulation tools. The novelty of the study lies in a systematic approach to the diagnosis and regulation of the stability of financial development. The systemic nature of the study is ensured by the application of the statistical framework of the System of National Accounts. Testing of the developed indicators and criteria was carried out in relation to the OECD+ (Organisation for Economic Co-operation and Development) countries for the period 2007–2020. As a result of the testing, a rating of countries was built according to the level of stability of financial development. This rating highlighted leading countries as well as countries generating systemic risks. The study also assessed the legal grounds behind the introduction of a countercyclical markup by a number of countries. The new diagnostic and regulatory system has a number of advantage—it is highly representative and objective by nature, and has a wide range of applications. The use of this system will improve the complexity and quality of diagnosing and regulating the stability of financial development at the national and global levels, as well as increase the effectiveness of public decision-making.

## Introduction

A scientific and practical interest in the problems of financial development of countries has been growing recently. In part, this happened because by solving these problems the countries can provide their national economies with their own financial resources, obtain additional income from the export of financial resources to other countries, and counter the threats of global financial risks. This is evidenced by the increase in the number of strategic plans for the financial development of countries [1–13] and the number of scientific publications [14–29] on this topic. However, an analysis of strategic plans shows that their main goal is to develop the financial sector of national economies without taking into account the systemic risks of this development. Meanwhile, as noted in a number of publications [30], excessive growth of the financial sector can provoke a financial crisis.

This situation is largely due to the lack of international standards for diagnosing and regulating the stability of financial development. International financial institutions, including the International Monetary Fund, only identify certain areas of analysis and propose an indicative list of indicators that should be taken into account when introducing regulatory measures [31]. Because of that, central banks are forced to develop independent indicators and criteria for diagnosing and regulating the stability of financial development. This leads to the emergence of different methodological constructs that reflect the specifics of the development of individual countries [32]. The conclusions drawn from these designs make it difficult to compare stability, develop appropriate public policies and coordinate these policies among countries.

Problems in the identification and regulation of the stability of financial development have attracted the attention of the scientific community. However, the analysis of scientific publications on this topic indicates a number of unresolved problems. In particular, there is still no consensus on understanding the stability of financial development and defining universal indicators to measure and assess it at the global level of the world economy. The problem with appropriate identification of global risks to financial stability, as well as the boundaries and timing of supranational and state intervention in market processes, remains unresolved. The desire to find answers to these questions motivated the present study.

The aim of the study is to develop a methodology for diagnosing and regulating the stability of financial development based on the use of a systematic approach.

In order to achieve this objective, it was hypothesized that the methodology for developing international requirements for diagnosing and managing the stability of a country’s financial development should be based on the principles of a systematic approach, which could be implemented by using the statistical framework of the System of National Accounts (SNA), which reflects all financial flows and their interrelationships at the national and global levels of the world economy.

To prove this hypothesis, the study developed indicators and criteria for quantitative and qualitative assessment of the stability of the financial development of countries; built a ranking of countries by the level of stability of financial development; identified the leading countries in this field; developed a "risk map" of the stability of financial development at the global level of the world economy; identified sources of risks of financial development in the form of countries with a low level of stability, which require the introduction of countercyclical measures. The results obtained were verified for adequacy by comparing the conclusions of the study with the practice of central banks to introduce a countercyclical markup, as well as with the results of similar studies performed by other authors.

## Literature review

Analysis of the results of research in the field of diagnostics and regulation of financial stability at the national and global levels of the world economy shows the following.

First, most of the publications focus on diagnosing and regulating the stability of financial markets and the stability of financial systems. Publications directly related to the topic of financial stability are extremely rare. As follows from the publications [33, 34], this may be due to the uncertain scientific status of the concept of financial development and the fragmentary nature of the metrics that signal an excessive accumulation of systemic risks.

Second, research on the stability of financial markets is carried out mainly in two directions: on the basis of interest rate analysis [30, 35] and on the basis of identification of the credit cycle phase [32, 36–40]. At the same time, within the framework of the second direction, models for assessing the cyclic components of the credit GAP or its transformations, as well as early warning models are distinguished. Recommendations for the use of alternative statistical filters occupy an important place in the study of the cyclic component [41, 42].

Third, studies of the stability of financial systems are dominated by the analysis of the stability of the banking sector. This analysis is carried out both on the basis of already known indicators (Z-score) and by developing stress tests [43].

Fourth, studies of the effectiveness of regulatory measures used to smooth cycles mostly revolve around attempts to determine the signal points for the introduction of a countercyclical markup (CM) [44–47]. At the same time, the research carried out is fragmentary and is usually limited to a narrow segment of lending, which is targeted by macroprudential measures. According to some authors [48], this approach to determining signal points is not reliable enough, since the dynamics observed in the analyzed segment can be compensated by the dynamics of other segments.

Fifth, studies of financial stability are mainly based on sample observations. Samples generally include individual countries or small groups of such countries. Such an approach implies the use of different indicators, criteria and algorithms that reflect the specifics of the macroeconomic conditions, the organizational structure of the economy, the scale of financial activities, values and goals of countries, and thus making it difficult to conduct a comparative analysis and identify the sources of generation of systemic risks.

Based on the above, it can be concluded that the studies carried out to date differ in terms of how they understand, measure, and assess the stability of financial development. In addition, these studies are based on a segmental approach to objects and subjects of diagnostics and regulation of the stability. This segmentation manifests itself in the analysis of the credit market, mainly in terms of identifying the excessive debt of non-financial corporations and individuals; in the analysis of the banking sector of the economy, rather than the analysis of all sectors of the economy; in the analysis of the financial stability of individual countries or their small groups. Such a segmentation is dangerous because it does not allow to form a general idea of the state and dynamics of financial development stability at the global level of the world economy, which can lead to contradictory conclusions and recommendations to public authorities.

In this regard, there is a need to develop universal and more representative indicators and criteria that allow conducting a full analysis of the stability of the financial development of countries, ranking countries by the level of achieved stability, identifying the best practices in managing the stability of financial development, and highlighting countries that pose potential threats to financial stability at the global level of the world economy.

## Methods

The study is based on the application of a systematic approach to the diagnosis and regulation of the stability of the financial development of countries. This approach will be adapted to the purpose of the study by applying the statistical framework of the System of National Accounts 2008 [49], which includes a comprehensive, systematic, and flexible set of macroeconomic accounts designed to analyze the world economy at the global and national levels, as well as for public policy and decision-making.

The study uses statistics from 39 countries that are current and potential members of OECD (OECD+). The list of analyzed countries and the period of analysis were formed taking into account the availability and representativeness of official data, as well as the identity of the units of measurement of these data. The statistics for the empirical study are taken from account №.720. Financial accounts—non consolidated—SNA 2008 for the period 2007–2020 [50].

The following assumptions were made in the study:

1. The ratio of the total financial assets of these countries to the population is used as an indicator of the financial development of countries. This choice is explained by the higher representativeness of the data and the higher resistance of the indicator "Population size" to changes in macroeconomic conditions, compared with the indicator "Gross domestic product" [33];
2. Identification of the stability of financial development is carried out on the basis of the identification of the cyclic component by calculating the amplitude of fluctuations of financial development indicators. This approach to assessing the stability of financial development is explained by the dynamic nature of development processes.

### Indicators of stability of financial development of countries

Taking into account the assumptions made, the indicator “Financial development index growth rate” is used to determine the dynamics of financial development of countries. This indicator is calculated by the formula (1):

$$GR_{FDI}=I_{n}/I_{n-1}-1$$ (1)

where:

*GR*_*FDI*_–the growth rate of the financial development index,

I–Financial Development Index,

n–is the period number.


I=FA/P (2)

where:

FA–financial assets of the domestic economy and the rest of the world,

P–the population of the country.

### Assessment of the cyclical component of the stability of the financial development of countries

The calculation of the cyclic component in the dynamics of financial development is carried out on the basis of the ratio of actual and equilibrium processes that allow determining the excessive accumulation of risks:

$$I_{SFDi}=GR_{FDIi}-TGR_{FDIi}$$ (3)

$$TGR_{FDIi}=a\cdot i+b$$ (4)

where:

*I*_*SFD*_–Financial Development Stability Index,

*TGR*_*FDI*_–trend of the growth rate of the financial development index,

*a*, *b*–the parameters of the trend equation,

i–year number,

n–the number of observation periods.

### Identification of risk areas in the financial development of countries

To determine the excess accumulation of risks in the financial sector of the world economy, it is proposed to use the criteria presented in Table 1. The criteria are modular values of *I*_*SFD*_ indicators divided into 5 intervals. These intervals correspond to the levels of stability of financial development: high, above average, average, below average, low. The quantitative values of the criteria should be established in accordance with the recommendations of the international financial organizations (Basel III) in the field of counter-cyclical regulation of the financial development of national economies. According to these recommendations, it is proposed to introduce a countercyclical markup when the GAP values increase from 2 to 10 percentage points [51].

**Table 1:** Criteria for financial stability.

Stability level	Criteria	Risk zones	Regulatory Zones
High	0≤ |ID| < _k1_	Risk-free zone	No need for countercyclical measures
Above average	_k1_ ≤ |ID| < _k2_		
Average	_k2_ ≤ |ID|< _k3_	Neutral zone	
Below average	_k3_ ≤ID< _k4_	Risk zone	Need for countercyclical measures
Low	_k4_ ≤ ID		

Source: authoring.

The resulting ranges of financial stability are put in line with the risk zone: the risk-free zone (the stability level is "high" and "above average"), the neutral zone with the stability level is "average" and, the risk zone (the stability level is "below average" and "low"). Risk zones, in turn, form regulatory zones.

In the study, it is proposed to distinguish two regulatory zones: requiring and not requiring the introduction of countercyclical measures. Wherein the regulatory zone requiring the introduction of countercyclical measures corresponds to the risk zone; and the regulatory zone not requiring the introduction of countercyclical measures includes a risk-free and neutral zone.

**Fig 1:**
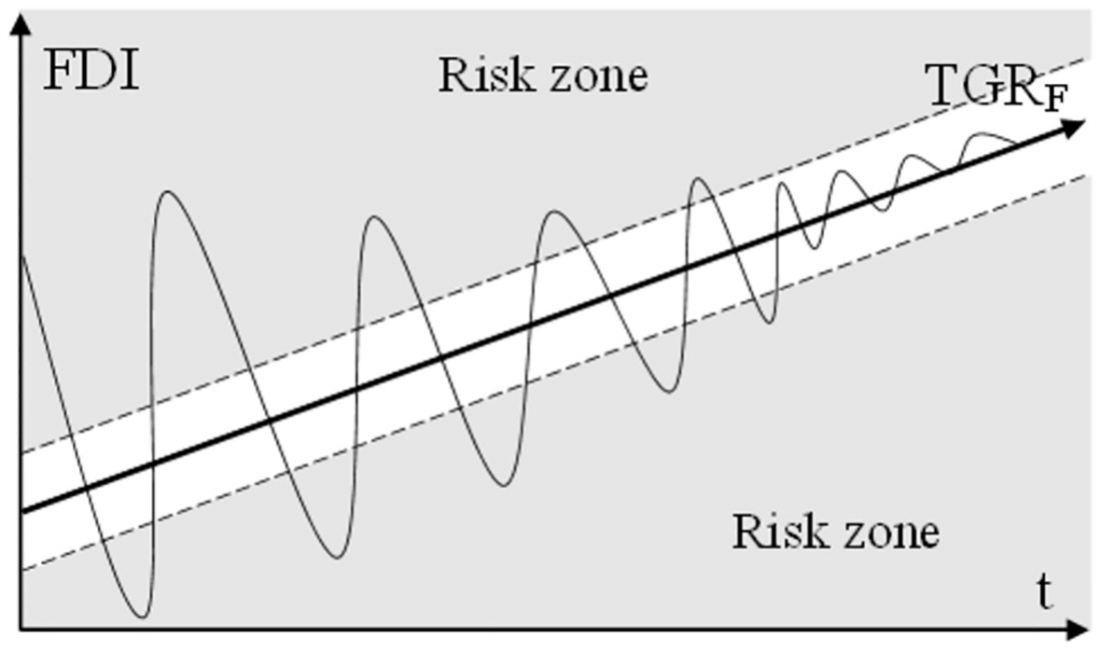
Regulatory zones (risk zones). Source: authoring.

Graphical interpretation of regulatory zones is shown in Fig 1.

Fig 1, two regulatory zones are highlighted. The first regulatory zone is an area with an acceptable level of risk. It includes neutral and risk-free zones (Table 1). This regulatory zone characterizes the desirable range of financial stability. The second regulatory zone is characterized by excessive accumulation of risks and therefore indicates the need to introduce regulatory measures to reduce the level of risks. Locating a country at risk is a reason for imposing countercyclical measures in that country. These measures are divided into two groups: proactive measures (countercyclical markup) and rapid response measures (markups on risk factors). Due to the proactive nature of the countercyclical markup, it is desirable to adopt it when the actual values of the indicator are in the lower risk zone. Rapid response measures should be used when the actual values of the indicator are in the top risk zone.

## Results

Testing of the developed indicators and criteria for assessing the stability of financial development was carried out for current and potential OECD members (OECD+). The list of these countries includes 39 countries. Statistical data for testing were taken from the data of the invoice No. 720. Financial accounts—non consolidated—SNA 2008 for the period 2018–2019 [50].

On the basis of these statistics, the growth rates of the financial development indices of the analyzed countries (*GR*_*FDI*_) for the period 2008–2020 were calculated and trends were constructed *TGR*_*FDI*_.

A graphical representation of the obtained results is shown in S1 File. Visual analysis of the charts shows a number of asymmetries in the financial development of countries.

First, countries are distinguished by the nature of trends that show the direction of financial development. In particular, the trend in Japan characterizes the invariability of financial development. The downward trend is observed in Brazil, Chile, Colombia, Korea, Luxembourg, New Zealand, Russia. Upward trend—in Austria, Belgium, Canada, Czech Republic, Denmark, Estonia, Finland, France, Germany, Greece, Hungary, Iceland, India, Ireland, Israel, Italy, Latvia, Lithuania, Mexico, Netherlands, Norway, Poland, Portugal, Slovak Republic, Slovenia, Spain, Sweden, Switzerland, Turkey, UK, USA. At the same time, the upward nature of trends is most pronounced in such countries as Denmark, Estonia, Hungary, Ireland, Israel, Lithuania, USA. From this analysis, it can be concluded that due to the accelerated financial development Denmark, Estonia, Hungary, Ireland, Israel, Lithuania, USA, are likely to become sources of risk for the stability of financial development on a global scale in the future.

Second, countries have different volatility of the financial development. Denmark, Estonia, Israel, Korea, Latvia, Lithuania, Netherlands, Russia, Slovak Republic, Slovenia, Spain, Sweden, Switzerland are the most volatile. In contrast, in Chile, Colombia, France, Iceland, New Zealand, UK, USA, the deviation of actual values from trending is minimal. This indicates that the first group of countries is likely to be a source of risk for the stability of the financial development of the world economy.

Third, countries have shown different responses to crises. Thus, in 2008, the maximum negative value of the stability index (*I*_*SFD*_) was observed in Chile (-26.77), Iceland (-23.79), New Zealand (-23.43), Canada (-21.69); and the maximum positive value was observed in Japan (13.21), Hungary (13.08), Slovak Republic (12.67). In 2014, the maximum negative value of the stability index was observed in Russia (-31.29) Greece (-15.26), Czech Republic (-14.41); and the maximum positive value was observed in Luxembourg (8.3), Turkey (5.81) India (2.36). During the Pandemic of 2020, Mexico (-3.65), Luxembourg (-0.12) had a negative stability index; and Hungary (22.56), Greece (16.74), Lithuania (15.11) had a maximum positive stability index.

Fourth, the graphs show the different effectiveness of the regulation of the financial development of countries. The decrease in volatility by the end of the analyzed period indicates an increase in regulatory efficiency. And on the contrary, an increase in volatility indicates a decrease in efficiency. An example of increased regulatory efficiency is Canada, Chile, Luxembourg, Mexico, New Zealand, Norway. An example of reduced efficiency is Hungary, India, Israel, Lithuania, Spain.

**Fig 2:**
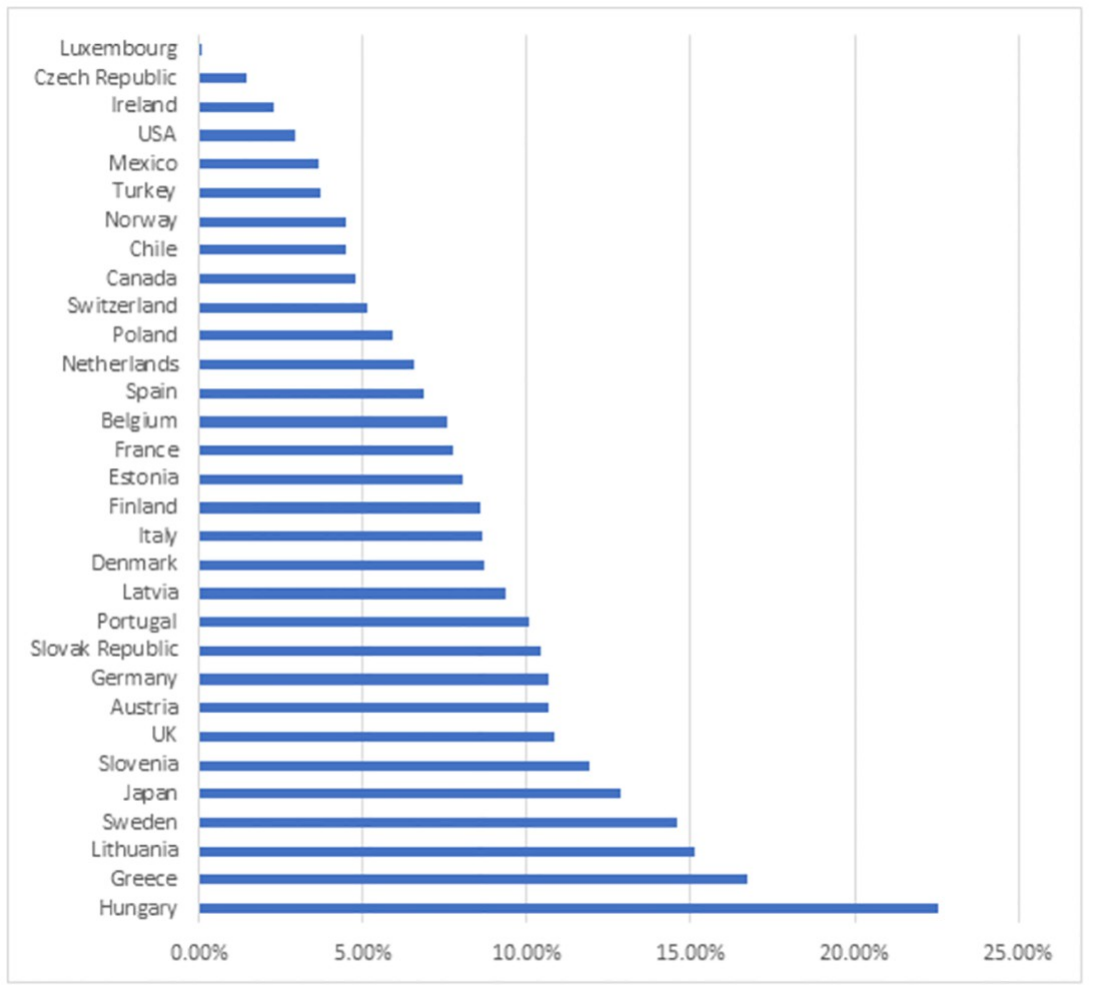
Rating of countries on the stability of financial development in 2020. Source: authors’ calculations based on the official statistical data [50].

Based on the indices of stability of financial development, the ranking of countries in 2020 was built (Fig 2). When building the rating, the criterion was applied, according to which the best values of financial stability correspond to the minimum values of the indices (*I*_*SFD*_), and the worst—to their maximum values.

The analysis of the rating of countries showed the following. First, the top five countries in terms of stability of financial development in 2020 included Luxembourg (0.12), Czech Republic (1.46), Ireland (2.32), USA (2.95), Mexico (3.65). Second, Japan (12.85), Sweden (14.56), Lithuania (15.11), Greece (16.74), Hungary (22.56) were at the lowest places in the ranking. Third, there is a significant gap in the level of stability of the financial development of the leading country (Luxembourg) and the country that closes the rating (Hungary). It is more than 22 p.p.

To determine the excess accumulation of risks in the financial sector of the world economy, a "Risk Map" was developed dividing countries into five levels of stability: high, above average, average, below average, low (Table 2).

**Table 2:** Levels of financial stability of OECD+ countries.

Stability level	Criteria, pts.
High	0≤ |ID| <2.0
Above average	2.0≤ |ID| < 4.0
Average	4.0≤ |ID| <6.0
Below average	6.0≤ |ID| < 8.0
Low	8.0≤ |ID|

Source: authoring.

Stability levels were formed in steps of 2 points. The step size was selected based on the following considerations.

The minimum value of the criterion (|ID|), equal to zero, characterizes the coincidence of the actual value of the financial stability index with its equilibrium value. The maximum value of the criterion (|ID|) equal to 8.0 pts characterizes the lower limit of the "low level" stability range. According to the recommendations of Basel III and the practice of their application in different countries, the deviation of the actual values of the cyclic component from the equilibrium level for a relatively long period of time (at least 4 quarters) in the amount of 2 p.p. is a signal for the introduction of a countercyclical markup. Therefore, the annual deviation of the cyclic component from the equilibrium level will be 8.0 pts. Based on the difference in the minimum limit values of the indicators and the number of intervals within these limit values (4 intervals), the step value in the "Risk Map" will be 2%.

Using the Risk Map (Table 2) a comparative analysis of the stability of financial development of OECD+ countries for 2018–2020 was carried out. The results of this analysis are presented in Table 3.

**Table 3:** Stability levels of financial development of OECD+ countries in 2018–2020, US$ thousands.

Stability level	2018	2019	2020
**High**	Colombia (0.09); Japan (1.37); Mexico (1.38)	Sweden (0.35); Italy (0.63); Netherlands (0.66); Iceland (0.74); France (0.75); Belgium (0.75); Finland (0.91); Colombia (1.07); Chile (1.39); Germany (1.43); Slovenia (1.71); Denmark (1.81); Austria (1.83); Estonia (1.84); Greece (1.92)	Luxembourg (0.12); Czech Republic (1.46)
**Above average**	Finland (2.3); Korea (3.02); New Zealand (3.22)	Portugal (2.02); Ireland (2.32); New Zealand (2.35); Japan (2.41); Switzerland (2.49); Poland (2.65); UK (2.93); USA (2.97); Spain (3.14); Luxembourg (3.45); Norway (3.95)	Ireland (2.32); USA (2.95); Mexico (3.65); Turkey (3.7)
**Average**	Russia (5.8); Slovak Republic (5.91)	Slovak Republic (5.15); Latvia (5.34)	Norway (4.48); Chile (4.5); Canada (4.79); Switzerland (5.16); Poland (5.94)
**Below average**	Portugal (6.3); Slovenia (6.51); India (6.56); Brazil (6.89); Spain (7.68)	Mexico (6.28); Czech Republic (6.38); Hungary (6.4); Canada (6.49); Lithuania (7.54); Turkey (7.84)	Netherlands (6.56); Spain (6.85); Belgium (7.6); France (7.77)
**Low**	Norway (8.18); Austria (8.45); USA (8.47); Greece (8.55); Poland (9.17); Ireland (9.29); Italy (9.43); Czech Republic (9.5); Germany (9.61); Switzerland (9.73); UK (9.77); Netherlands (10.19); Latvia (10.85); Chile (11.13); Belgium (11.43); France (11.6); Estonia (11.9); Luxembourg (12.27); Canada (12.83); Lithuania (12.86); Denmark (13.66); Iceland (13.76); Israel (14.15); Sweden (16.4); Turkey (17.16); Hungary (20.56)	Israel (8.46); Korea (11.58)	Estonia (8.03); Finland (8.61); Italy (8.66); Denmark (8.68); Latvia (9.33); Portugal (10.09); Slovak Republic (10.43); Germany (10.66); Austria (10.68); UK (10.86); Slovenia (11.89); Japan (12.85); Sweden (14.56); Lithuania (15.11); Greece (16.74); Hungary (22.56)

Source: authors’ calculations based on the official statistical data [50].

As seen from Table 3, in 2018, only three countries had a high level of stability of financial development: Colombia; Japan; Mexico. Low stability was observed in 27 countries: Norway; Austria; USA; Greece; Poland; Ireland; Italy; Czech Republic; Germany; Switzerland; UK; Netherlands; Latvia; Chile; Belgium; France; Estonia; Luxembourg; Canada; Lithuania; Denmark; Iceland; Israel; Sweden; Turkey; Hungary. In 2019, the stability of the OECD+ countries improved significantly. Fifteen countries achieved a high level of stability in financial development, and only Israel and Korea had a low level of stability. In 2020, as a result of the Covid-19 pandemic, the level of stability of financial development decreased. The number of countries with a high level of stability decreased to two (Luxembourg, Czech Republic), and the number of countries with a low level of stability increased to 17.

In general, an analysis of the dynamics of financial stability shows that OECD+ countries have become more resilient to crisis phenomena. This is largely due to tightening of regulatory measures on the part of central banks. In particular, during the period under review, many countries decided to introduce a countercyclical markup.

It is important to note that, contrary to the recommendations of Basel III, central banks imposed a countercyclical markup in the presence of a negative credit GAP. Thus, in Bulgaria, with a credit gap at (-42.7 pts) in September 2018, the countercyclical markup was increased to 0.5%. In Iceland, the credit gap in May 2018 amounted to (-76.9) pts, and the countercyclical markup was increased to 1.75%, in Ireland, with the credit gap at the level of (-77) pts in July 2018, the regulator decided to increase the countercyclical markup to 1%. Positive CM with a negative credit gap was also introduced in the UK, Denmark, Lithuania, Norway, and the Czech Republic [32].

At the same time, it should be noted that the use of the criteria developed in this study avoids contradictions between the methodology and the practice of introducing a countercyclical markup. This is evidenced by the data in Table 4.

**Table 4:** A comparative analysis of the criteria and practices for the introduction of a countercyclical allowance.

Country	Stability index in 2018	Recommended* increase in the level of AN allowance from 2019 (+)	Actual increase in AN level in the period 2019–2020.		Coincidence Not Match
			Level	Effective Introduction Date	
Denmark	-13.66	**+**	0.5	31.03.2019	+
			1.0	30.09.19	
Iceland	-13.76	**+**	1.75	15.05.2019	+
Lithuania	-12.86	**+**	0.50	01.01.2019	+
			1.0	30.06.2019	
Norway	-8.18	**+**	2.5	31.12.2019	+
Slovakia	-5.91	**-**	1.5	01.08.2019	-
Sweden	-16.4	**+**	2.5	01.01.2020	+
Great Britain	9.77	**-**	0	-	+
Russia	-5.8	**-**	0	-	+
Ireland	-9.29	**+**	1.0	05.07.2019	+
Luxemburg	-12.27	**+**	0.25	01.01.2020	+
France	-11.6	**+**	0.25	01.07.2019	+
			0.5	02.04.2020	
Czech Republic	-9.5	**+**	1.25	01.01.2019	+
			1.5	01.07.2019	
			1.75	01.01.2020	
Switzerland	-9.73	**+**	0	-	-

Compiled based on the information of the European Systemic Risk Board [52].

* recommended by the results of this study.

As seen from Table 4, according to the developed criteria, the countercyclical markup should have been introduced in 10 countries out of 13 countries. In fact, 10 countries have also decided to introduce a countercyclical markup. However, despite the quantitative convergence of the total number of countries, there are differences between the two countries. In particular, according to the developed criteria, a countercyclical markup should not have been introduced in Slovakia. The decision of the central bank of this country can be explained by the fact that the stability index came very close to the border of regulatory measures (6.0 pts). On the contrary, according to the developed criteria, Switzerland should have introduced a countercyclical markup, but this was not done. As a result, Switzerland’s stability index declined to (-10.86 pts) in 2020. Thus, the application of the developed criteria makes it possible to bring the signal points for the introduction of regulatory measures in line with the practice of decision-making by central banks.

## Discussion

As a result of the study, new indicators have been developed to diagnose the financial development of countries. The advantage of the indicators and criteria developed is that they are more representative, as they cover the full range of internal and external financial flows reflected in the System of National Accounts. Increased representativeness allows for more accurate measurement of countries’ financial stability and the identification of additional risks not accounted for by central banks.

The criteria proposed in the study for diagnosing and regulating the stability of financial development make it possible to form "Risk maps" on a global scale and to identify regulatory zones that signal the need for countercyclical measures. At the same time, the rule that regulatory measures must be differentiated depending on the positive and negative values of the financial stability index is highlighted. This approach to regulating the stability of financial development is proposed for the first time and makes it possible to harmonize the methodology and practice of introducing countercyclical regulatory measures by central banks. Moreover, the “signal points” identified on the basis of this approach are universal, i.e. applicable to all countries at the same time. This makes it possible to consider them as a platform for the development of international standards in the field of diagnostics and regulation of stability of financial development.

The conclusions obtained as a result of the study supplement the results of the study [44], explaining the low effectiveness of the Basel gap, used as a key reference for the introduction of countercyclical measures. The study also confirms the conclusions [35, 45] that the proactive use of countercyclical capital regulation can help mitigate boom and bust cycles caused by over-optimistic expectations.

It should be noted that the study is the first step towards standardizing the requirements for a full-scale system for measuring and assessing the stability of countries’ financial development, allowing to identify the sources of global risks (countries with a low level of stability) and, on this basis, to improve regulatory requirements. Further steps in this direction may be associated with the following tasks.

First, the study suggests that the financial development index should be measured in relation to population rather than GDP. This is due to the fact that the population, in comparison with GDP, is an indicator that is more resistant to crisis phenomena and does not contain methodological discrepancies in its calculations. At the same time, this indicator can be refined by using not the entire population, but its economically active part.

Second, the study analyses the time series of a country’s financial assets on the basis of annual data. Meanwhile, data on the magnitude of financial assets are published by central banks on a quarterly basis. An analysis of the stability of financial development on the basis of quarterly data would clarify the results obtained in this study.

Third, the study did not use filters applied by central banks and individual authors of scientific publications in calculating the equilibrium level of financial stability of countries. This is due to the fact that filters are generally used in the analysis of data with a higher measurement frequency. However, the use of filters to determine the cyclic component based on quarterly data is a promising area for further research.

Fourth, the study calculated the criteria for the formation of levels of stability of financial development of countries in relation to OECD+ countries. To use the new system of indicators on a global scale, the calculation of the criteria needs to be clarified. Doing this requires an analysis of financial assets for all countries of the world or for a representative sample of these countries.

Fifth, a new system of indicators and criteria has been developed and tested in relation to a common indicator of the stability of a country’s financial development. An analysis of stability in the context of financial instruments and sectors of the economy could be a good complement to the study and would allow obtaining a better visual representation of the formation of systemic risks in the financial sector of the world economy.

## Conclusions

Measuring, asessing, and regulating the stability of the financial development of countries is a very relevant and relatively new direction in studies of financial stability. The peculiarity of the research carried out in this area is their fragmented nature, which does not allow for the development of international standards for diagnosing and regulating the stability of financial development on the basis of the existing methodological framework. In this regard, the purpose of this study is to develop a methodology for diagnosing and regulating the stability of financial development based on a systematic approach.

As a result of the study, new indicators have been developed to diagnose the financial development of countries. They are based on a systematic approach using SNA data on countries’ financial assets. Simultaneous accounting of financial flows (internal and external) across the spectrum of financial instruments is a new direction in measuring financial development and its stability. The developed indicators allow for a comparative analysis of stability and the formation of ratings of countries on the level of stability of financial development. For example, a comparative analysis of the stability of the financial development of OECD+ countries made it possible to establish the following.

First, countries are distinguished by the nature of trends that show the direction of financial development. At the same time, the upward nature of trends is most pronounced in such countries as Denmark, Estonia, Hungary, Ireland, Israel, Lithuania, USA. In view of accelerated financial development, these countries are likely to become sources of risk for global financial stability in the future.

Second, countries’ financial development has different volatility. Denmark, Estonia, Israel, Korea, Latvia, Lithuania, Netherlands, Russia, Slovak Republic, Slovenia, Spain, Sweden, Switzerland are the most volatile. This shows that this group of countries is a source of risk for the stability of the financial development of the world economy.

Third, countries have shown different responses to crises. Thus, for example, in the pandemic 2020, a negative value of the stability index was observed in Luxembourg and Mexico, and the maximum positive value was observed in Greece, Hungary, Lithuania.

Fourth, the OECD+ countries differ in their effectiveness in regulating the stability of financial development. An example of increased regulatory efficiency is Canada, Chile, Luxembourg, Mexico, New Zealand, Norway. An example of reduced efficiency is Hungary, India, Israel, Lithuania, Spain.

The ranking of countries and its analysis showed that in 2020, Luxembourg, the Czech Republic, Ireland, USA, Mexico were the leaders in financial stability. Japan, Sweden, Lithuania, Greece, Hungary took the lowest positions in the ranking. Thirdly, there is a significant gap in the level of stability of the financial development of the lead country (Luxembourg) and the country that closes the rating (Hungary). It is more than 22 pts.

The developed criteria made it possible to form "Risk Maps", "Risk Zones" and "Regulatory Zones" for OECD+ countries, as well as to determine the signal points and the time of introduction of countercyclical measures, taking into account their proactive and tactical nature. This part of the study revealed additional sources of risk not accounted for by central banks. In particular, a countercyclical markup should have been introduced in Switzerland starting in 2019. Due to the fact that the central bank of this country did not make a decision, in 2020 the level of stability of financial development in Switzerland decreased by 1.13 pts compared to 2018.

In general, testing of the new indicator system and criteria confirmed their applicability in practice and made it possible to harmonize the methodology and practice of decision-making on countercyclical regulation.

## Supporting information

S1 File Growth rates of the countries’ financial development index. Source: authors’ calculations based on the official statistical data [50].
(RAR)
